# Plasma α-synuclein and QSM-derived susceptibility in Parkinson’s disease: associations with clinical features

**DOI:** 10.3389/fnagi.2026.1740309

**Published:** 2026-08-14

**Authors:** Xingjun Li, Tianfei Luo, Jing Bai

**Affiliations:** Neuroscience Center, The First Hospital of Jilin University, Changchun, China

**Keywords:** biomarkers, brain iron deposition, clinical correlation, neuroimaging, Parkinson’s disease, quantitative susceptibility mapping (QSM), α-synuclein

## Abstract

**Background:**

The pathological interplay between α-synuclein aggregation and iron dysregulation is central to the neurodegenerative process in Parkinson’s disease (PD). Quantitative susceptibility mapping (QSM) allows *in vivo* assessment of regional brain iron burden, and blood assays enable measurement of plasma α-synuclein; however, their clinical relevance and mutual relationship remain insufficiently defined.

**Objective:**

To evaluate plasma α-synuclein as a diagnostic biomarker (against controls) and to explore whether regional brain iron deposition, measured by QSM, correlates with clinical features and plasma α-synuclein within the PD group.

**Methods:**

Fifty-two PD patients and sixty-two age- and sex-matched healthy controls were enrolled. Plasma α-synuclein concentrations were quantified by ELISA. Magnetic susceptibility values of seven deep gray-matter nuclei were obtained using QSM reconstructed from a multi-echo gradient-echo sequence on a 3.0 T MRI scanner. Motor and non-motor symptoms were assessed using MDS-UPDRS-III, H-Y staging, MoCA, MMSE, HAMD, and HAMA scales.

**Results:**

Plasma α-synuclein levels were significantly higher in PD than controls [median 54.9 (46.9–60.6) vs. 28.7 (24.4–33.7) pg/μL, *p* < 0.001]. ROC analysis showed strong diagnostic accuracy (AUC = 0.976, sensitivity = 100%, specificity = 88.7%). Within PD, plasma α-synuclein correlated inversely with Age (*r* = −0.371, *p* = 0.007) but not with other clinical parameters. In unadjusted analyses, regional QSM values showed nominal associations with motor severity and cognitive performance; however, these associations did not remain significant after adjustment for age and multiple comparisons.

**Conclusion:**

Elevated plasma α-synuclein provides a highly discriminative diagnostic biomarker for PD, while regional iron accumulation measured by QSM-derived magnetic susceptibility exhibited nominal associations with clinical and cognitive measures, although these relationships did not remain significant after age adjustment and correction for multiple comparisons. The absence of a direct association between peripheral α-synuclein levels and regional brain iron burden suggests that these biomarkers may reflect partially independent pathological processes in Parkinson’s disease. Future longitudinal studies are warranted to clarify the temporal dynamics and potential complementary roles of these biomarkers in disease characterization.

## 1 Introduction

Parkinson’s disease (PD) is the second most common neurodegenerative disorder, affecting approximately 1% of individuals over the age of 65 worldwide (Dorsey et al., 2007). Clinically, PD manifests through both motor symptoms—such as bradykinesia, rigidity, tremor, and postural instability—and non-motor features including cognitive decline, depression, anxiety, and sleep disturbances. Despite decades of investigation, the pathogenesis of PD remains incompletely understood, and reliable biomarkers for early diagnosis or disease monitoring are still lacking. Current evaluations rely primarily on clinical rating scales, which are subjective and insufficient for detecting early or subclinical alterations.

At the pathological level, PD is characterized by progressive dopaminergic neuronal loss within the substantia nigra and by intraneuronal inclusions of misfolded α-synuclein forming Lewy bodies (Luth et al., 2014; Ludtmann et al., 2018). α-synuclein, a presynaptic protein crucial for synaptic vesicle trafficking, undergoes conformational changes under pathological conditions, leading to oligomerization and fibrillation that exert neurotoxic effects (Venda et al., 2010; Colla et al., 2012). Mutations or overexpression of the *SNCA* gene accelerate α-synuclein aggregation and cause familial PD phenotypes (Lindersson et al., 2004; Kim et al., 2013; Durante et al., 2019). Moreover, misfolded α-synuclein can propagate between neurons, amplifying neurodegeneration through prion-like mechanisms, mitochondrial dysfunction, and neuroinflammatory cascades (Pemberton et al., 2011; Cascella et al., 2022; Gustot et al., 2015; Poewe et al., 2017). Consequently, abnormal α-synuclein accumulation represents not only a pathological hallmark but also a potential molecular biomarker of PD progression (Lassen et al., 2018; Brehme et al., 2014; Flavin et al., 2017).

Emerging evidence indicates that dysregulated iron metabolism also plays a critical role in PD pathophysiology (Aime et al., 2000; Costa-Mallen et al., 2017). Excessive iron deposition in the substantia nigra has been consistently demonstrated by histochemical and magnetic resonance imaging studies (Zeng et al., 2024; Costa-Mallen et al., 2015). Iron contributes to oxidative stress through Fenton chemistry, producing reactive oxygen species that trigger lipid peroxidation, mitochondrial injury, and ferroptotic neuronal death (Dixon et al., 2012; Mahoney-Sánchez et al., 2021). In parallel, α-synuclein interacts directly with iron and exhibits ferrireductase activity, converting Fe^3+^ to Fe^2+^ and thereby reinforcing oxidative toxicity (Urrutia et al., 2013; McCarthy et al., 2018). This bidirectional interaction forms a pathological feedback loop in which iron accumulation promotes α-synuclein aggregation, and aggregated α-synuclein further disturbs iron homeostasis (Dekens et al., 2021; Jeong and David, 2003; Belaidi et al., 2018). Understanding the interplay between systemic α-synuclein burden and regional brain iron load may therefore clarify key mechanisms of PD progression.

Quantitative susceptibility mapping (QSM) provides a non-invasive, *in vivo* method to provide a proxy for brain iron content by measuring tissue magnetic susceptibility derived from gradient-echo MRI (Wang and Liu, 2015). We caveat that QSM is not a direct quantification of iron, as the susceptibility signal also includes contributions from other sources such as myelin and calcium. QSM studies have revealed increased susceptibility in the substantia nigra, putamen, globus pallidus, and red nucleus of PD patients, correlating with disease severity and duration (Murakami et al., 2015; Lewis et al., 2018; Fu et al., 2021; Lancione et al., 2022). Concurrently, advances in peripheral biomarker assays, particularly enzyme-linked immunosorbent assay (ELISA), have enabled measurement of plasma α-synuclein as a minimally invasive indicator of neurodegenerative burden (Shu et al., 2024; Zubelzu et al., 2022). Nevertheless, reported findings remain inconsistent owing to methodological heterogeneity and disease-stage variability. Integrating QSM-based iron quantification with plasma α-synuclein profiling could thus yield complementary insights into the molecular and structural underpinnings of PD.

Based on this rationale, the present study aimed to investigate plasma α-synuclein concentrations and regional brain iron deposition measured by QSM in PD patients and healthy controls. We further assessed their relationships with detailed clinical indices encompassing motor and non-motor features. We hypothesized that plasma α-synuclein would be elevated in PD, QSM-derived magnetic susceptibility would correlate with disease severity, and combined evaluation of these biomarkers would provide a more comprehensive understanding of PD pathophysiology and clinical heterogeneity.

## 2 Materials and methods

### 2.1 Participants

This cross-sectional study enrolled 52 consecutive patients with idiopathic PD who attended the specialized PD outpatient clinic of the Neurology Department between August 2024 and January 2025, and 62 age- and sex-matched healthy individuals undergoing routine physical examinations during the same period. PD diagnosis followed the 2016 Chinese diagnostic criteria for primary PD (Chinese Medical Association, Neurology Branch, 2016) and the 2015 Movement Disorder Society (MDS) clinical diagnostic criteria (Postuma et al., 2015). All participants provided written informed consent, and the study protocol was approved by the institutional ethics committee.

#### 2.1.1 Inclusion and exclusion criteria

##### 2.1.1.1 PD group—inclusion criteria

(1) fulfilled both diagnostic standards above;

(2) able to undergo QSM MRI, peripheral blood sampling, and PD-related clinical scale assessments;

(3) absence of significant cardiac, hepatic, or renal dysfunction.

##### 2.1.1.2 PD group—exclusion criteria

Patients with Parkinson-plus syndromes or secondary parkinsonism; a history of psychiatric disorders or other neurological diseases; prior surgery or postoperative chemoradiotherapy for cancer; severe cardiopulmonary dysfunction; acute or chronic infectious diseases; refusal or non-cooperation during evaluation; anemia or iron-metabolism–related disorders; or contraindications to MRI.

##### 2.1.1.3 Control group—inclusion criteria

Age- and sex-matched healthy volunteers from routine physical examinations who provided informed consent and were able to undergo peripheral blood collection.

##### 2.1.1.4 Control group—exclusion criteria

History of PD, parkinsonism, essential tremor, Alzheimer’s disease, or dementia with Lewy bodies; history of psychiatric or other neurological disorders; or refusal/non-compliance during data collection.

### 2.2 Clinical and neuropsychological assessment

Collected variables included age, sex, disease duration, educational level (years), medical history, and clinical subtype. During baseline assessment in the medication “off” state, disease severity was rated using the MDS-UPDRS Part III (Goetz et al., 2008) and Hoehn–Yahr (H–Y) stage (Hoehn and Yahr, 1967). Cognitive function was assessed with the Montreal Cognitive Assessment (MoCA) and Mini-Mental State Examination (MMSE), while anxiety and depression were evaluated with the Hamilton Anxiety (HAMA) and Hamilton Depression (HAMD) scales, respectively.

Motor subtypes were determined using the MDS-UPDRS algorithm, defined as the average tremor score (items 2.10; 3.15a–b; 3.16a–b; 3.17a–e; 3.18) divided by the average axial score (items 2.12, 2.13, 3.10, 3.11, 3.12); ratios ≥ 1.15 indicated tremor-dominant (TD), ≤ 0.90 indicated postural instability/gait difficulty (PIGD), and 0.90–1.10 were classified as indeterminate (Goetz et al., 2008).

### 2.3 MRI acquisition and QSM analysis (PD group)

All PD patients underwent brain MRI on a 3.0 T Philips Ingenia MR scanner. Blood sampling, MRI acquisition, and clinical assessments were performed on the same day for each participant. The vast majority of patients were newly diagnosed and had not received any antiparkinsonian medication prior to enrollment; for the few patients who had been previously diagnosed and were on stable medication, assessments were conducted in the medication “off” state after a minimum 12-h washout period, consistent with the protocol used for clinical scale evaluations. Conventional T1-weighted images were acquired solely to exclude major structural abnormalities and to assist with anatomical orientation during ROI delineation; these images were not used for quantitative analysis.

QSM Acquisition Parameters: QSM data were acquired using a 3D multi-echo gradient-echo (ME-GRE) sequence with five symmetric echoes: TR = 50 ms, first echo time (TE1 ) = 5.6 ms, echo spacing (ΔTE) = 10.0 ms, and final echo time (TE5 ) = 45.6 ms. Other parameters included flip angle = 20°, FOV = 220 × 220 × 120 mm^3^, acquisition matrix = 220 × 220 × 80 slices, slice thickness = 1.5 mm, SENSE acceleration factor = 2, and total acquisition time = 6 min 9 s.

#### 2.3.1 QSM reconstruction pipeline

Phase and magnitude data from the multi-echo GRE sequence were processed in MATLAB R2016a using the STI Suite package. Complex images were first combined across receiver channels and a weighted least-squares fit across echoes was applied to estimate the local field map. Phase images were unwrapped using a Laplacian-based phase unwrapping approach. Brain masks were generated from the magnitude images using FSL BET. Background field contributions were removed using the RESHARP (regularization-enabled SHARP) algorithm. Finally, tissue magnetic susceptibility maps were reconstructed via iterative least-squares dipole inversion (iLSQR) as described by Wang and Liu (2015). Magnetic susceptibility values were expressed in parts per billion (ppb) relative to a reference region.

#### 2.3.2 ROI delineation

**FIGURE 1:**
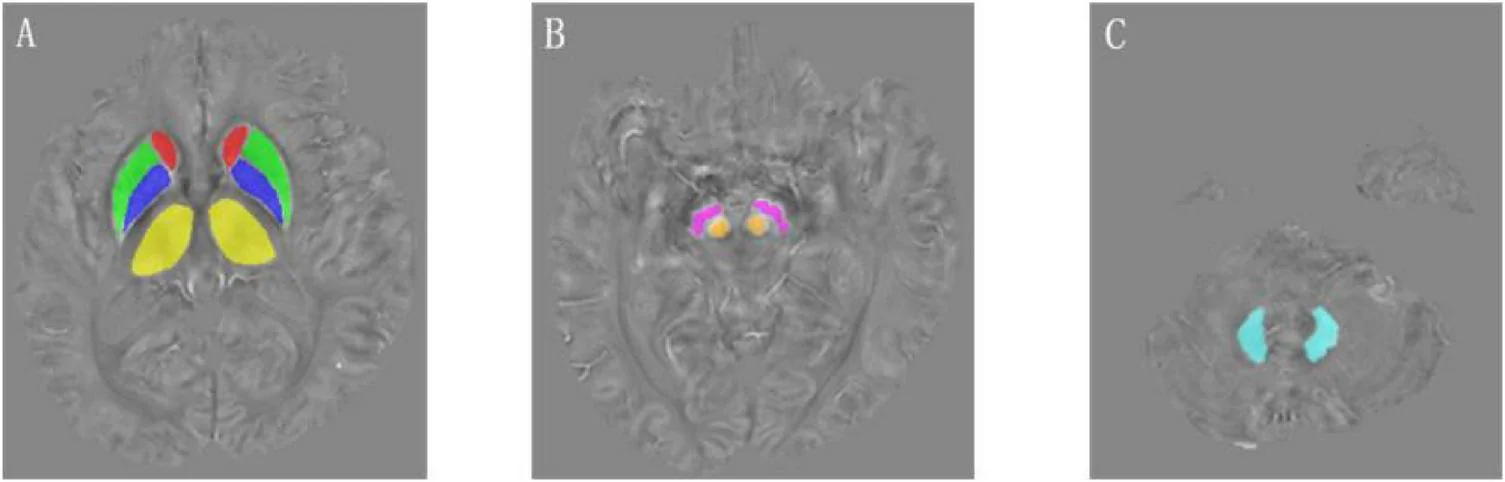
Shows the ROI delineation on QSM images. **(A)** Caudate nucleus (CN)—red; Putamen (Pu)—green; Globus pallidus (GP)—dark blue; Thalamus (Th) —yellow. **(B)** Red nucleus (RN)—orange; Substantia nigra (SN)—pink. **(C)** Dentate nucleus (DN) —light blue.

Regions of interest (ROIs) were manually delineated directly on the QSM images (susceptibility maps) using ITK-SNAP v3.6.0; T1-weighted images were used only for anatomical orientation when necessary, and no additional coregistration was performed. Two raters (one neuroradiologist and one neurologist), blinded to clinical data, independently drew bilateral ROIs for the caudate nucleus (CN), putamen (Pu), globus pallidus (GP), thalamus (Th), substantia nigra (SN), red nucleus (RN), and dentate nucleus (DN) (Figure 1). To minimize measurement error and subjectivity, for each structure, the four clearest and largest slices were selected; measurements were repeated twice and averaged per side, then across hemispheres and raters. Magnetic susceptibility was expressed in parts per billion (ppb), as described by Wang and Liu (2015).

### 2.4 Measurement of plasma α-synuclein

Plasma α-synuclein levels were measured using a commercially available ELISA kit (Human α-Synuclein Detection Kit, Hunan Taokang Maoyuan Biotechnology Co., Ltd., China National Medical Products Administration registration No. 湘械注准20222402002) following the manufacturer’s instructions. The kit employs a double-antibody sandwich ELISA format with horseradish peroxidase (HRP)-conjugated detection antibody and tetramethylbenzidine (TMB) substrate (Crowther, 2009). Briefly, plasma samples (50 μL) and provided controls were added to pre-coated microplates, incubated at 37°C for 60 min, washed five times with wash buffer, and then incubated with HRP-conjugated antibody (100 μL) at 37°C for 30 min. After washing, chromogenic substrates A and B (50 μL each) were added and incubated at 37°C in the dark for 15 min, followed by termination with stop solution (50 μL). Optical density (OD) was read at 450 nm within 15 min using a Tecan Infinite F50 microplate reader. All samples were assayed in duplicate. The intra-assay coefficient of variation (CV) was ≤ 15%, and the inter-assay CV was ≤ 20%, as specified by the manufacturer’s quality control specifications. OD values were converted to relative concentrations (pg/μL) using a laboratory-established standard curve generated from serially diluted positive controls. It should be noted that this kit is registered for clinical auxiliary diagnosis and we applied it to plasma samples collected with EDTA as anticoagulant, processed according to the manufacturer’s plasma preparation protocol (centrifugation at 3,500 rpm for 10 min).

### 2.5 Statistical analysis

Statistical analyses were performed using SPSS version 27.0. Normality was assessed using the Shapiro–Wilk test. Continuous variables were expressed as mean ± standard deviation (SD) for normally distributed data or median (interquartile range, IQR) for non-normal data. Group comparisons between PD patients and controls were conducted using independent-sample *t*-tests or Mann–Whitney U tests as appropriate. Categorical variables were compared using the χ^2^ test.

Correlation analyses were conducted in PD patients only. Raw associations were assessed using Spearman’s rank correlation coefficients. To account for the known influence of age on brain iron measures, age-adjusted partial Spearman correlations were performed for all analyses involving QSM measures and for correlations between QSM and plasma α-synuclein. Associations between plasma α-synuclein and clinical measures were also evaluated with age adjustment. Multiple linear regression analysis was further performed to evaluate the independent associations of plasma α-synuclein with clinical characteristics in the PD group.

To control for multiple comparisons across ROI-based analyses, false discovery rate (FDR) correction using the Benjamini–Hochberg procedure was applied separately for each family of tests. Both uncorrected *p*-values and FDR-adjusted *q*-values are reported. Statistical significance was defined as two-tailed *P* < 0.05.

Diagnostic performance of plasma α-synuclein was evaluated using receiver operating characteristic (ROC) curve analysis, with area under the curve (AUC), sensitivity, specificity, and 95% confidence intervals (CI) reported. Given the exploratory nature of subgroup analyses and the limited sample size, subtype-specific correlations were considered hypothesis-generating.

## 3 Results

### 3.1 General characteristics and plasma α-synuclein levels

A total of 114 participants were enrolled: 52 patients with PD and 62 healthy controls. The PD group had a median age of 65.50 years (IQR 62.00–71.00) with 18 males (34.6%) and 34 females (65.4%). The control group had a median age of 65.00 years (IQR 60.00–73.00) with 26 males (42.0%) and 36 females (58.0%). There were no significant between-group differences in age or sex (*P* > 0.05) (Table 1).

**TABLE 1:** Comparison of baseline characteristics and plasma α-synuclein levels between PD group and normal control group.

Item	PD group (*n* = 52)	Control group (*n* = 62)	*Z/χ^2^*	*P*
Age (years)	65.50 (62.00, 71.00)	65.00 (60.00, 73.00)	−0.350^a^	0.726
Gender/n (%)				
Male	18 (34.6)	26 (42.0)	0.639^b^	0.424
Female	34 (65.4)	36 (58.0)		
α-Synuclein concentration (pg/μL)	54.92 (46.97, 60.59)	28.65 (24.41, 33.71)	−8.690^c^	< 0.001***

^a^ Wilcoxon rank-sum test;

^b^ Chi-square test;

^c^ Wilcoxon rank-sum test.

***P < 0.001.

**FIGURE 2:**
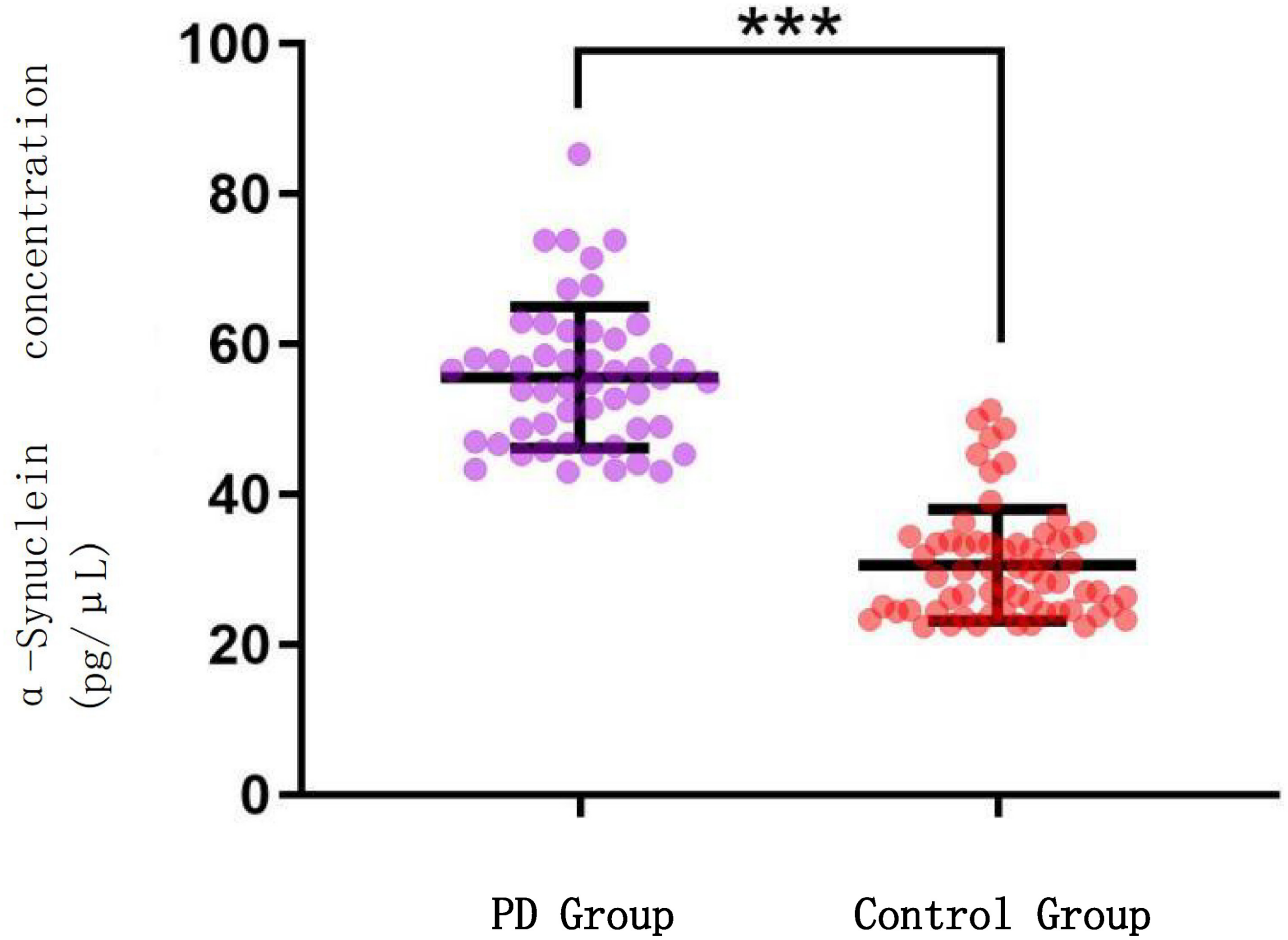
Plasma α-synuclein levels in PD group and normal control group. ***P < 0.001.

Plasma α-synuclein was significantly higher in PD patients [Median (IQR): 54.92 (46.97–60.59) pg/μL] than in controls [Median (IQR): 28.65 (24.41–33.71) pg/μL] (*Z* = −8.690, *P* < 0.001). Baseline characteristics and α-synuclein levels are presented in Figure 2 and Table 1.

**FIGURE 3:**
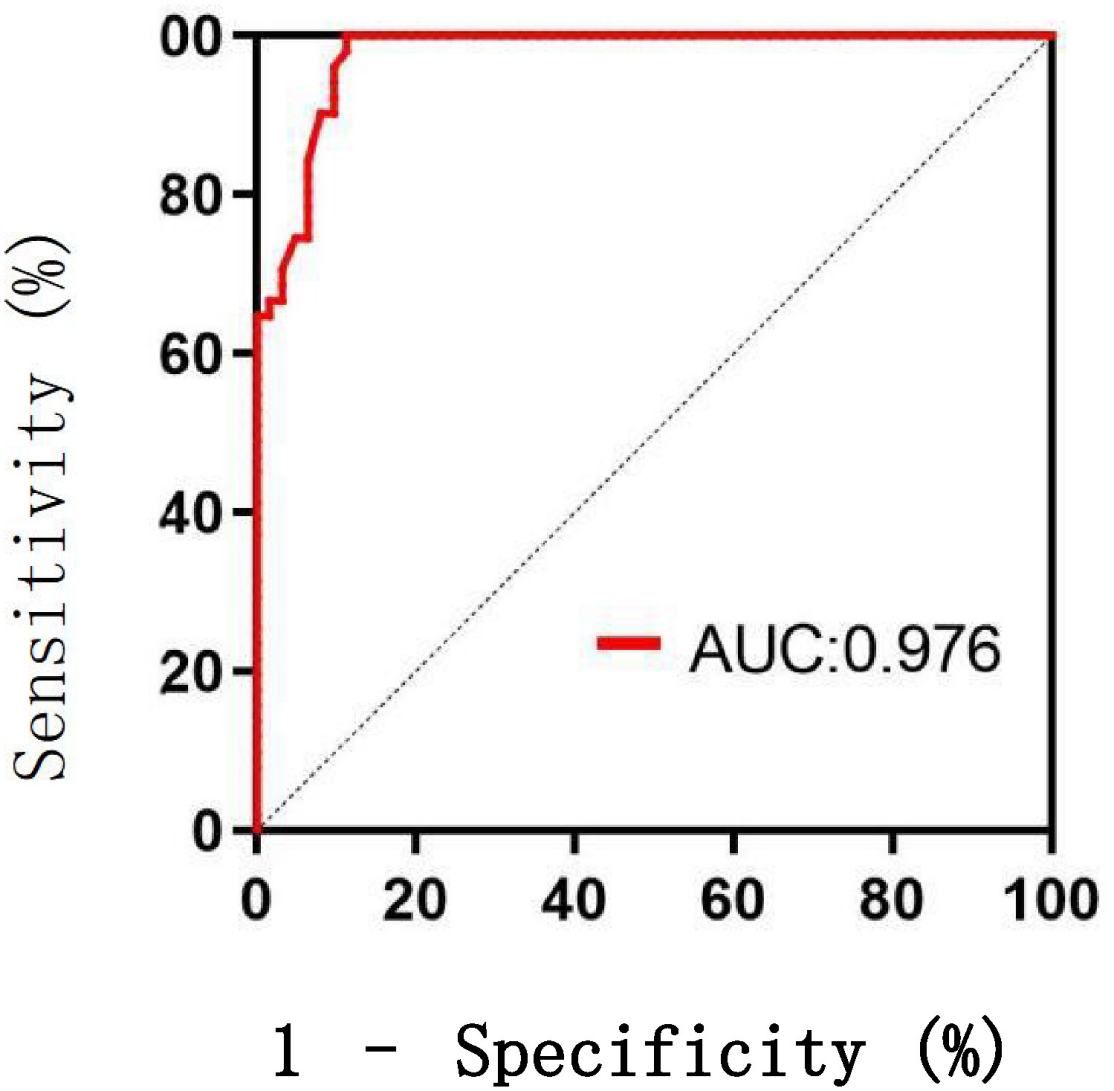
ROC curve of plasma α-synuclein for the diagnosis of PD.

ROC curve analysis showed AUC = 0.976 (95% CI 0.954–0.998, *P* < 0.001) with an optimal cut-off of 41.00 pg/μL, yielding 100.0% sensitivity and 88.7% specificity (Figure 3).

### 3.2 Correlations of plasma α-synuclein with clinical features (PD only)

In unadjusted Spearman analyses, plasma α-synuclein demonstrated a weak inverse correlation with motor severity assessed by MDS-UPDRS Part III (*r* = −0.284, *P* = 0.046). However, after adjustment for age, this association was no longer significant (*P* = 0.637) (Tables 2, 3).

**TABLE 2:** Correlation analysis between plasma α-synuclein levels and clinical characteristics in PD group.

Variable	*r*	*P*
Age	−0.371	0.007*
Disease duration	−0.110	0.451
MDS-UPDRS III	−0.284	0.046*
Hoehn–Yahr stage	−0.244	0.088
MoCA	−0.165	0.261
MMSE	−0.117	0.430
HAMA	−0.089	0.544
HAMD	−0.112	0.445

*r* = correlation coefficient;

*P < 0.05.

**TABLE 3:** Age-adjusted associations between plasma α-synuclein and clinical characteristics in Parkinson’s disease.

Clinical variable	Age-adjusted r	*P*-value	FDR *q*-value
Disease duration	−0.1	0.45	0.78
MDS-UPDRS III	0.07	0.64	0.78
H-Y	−0.18	0.21	0.78
MoCA	−0.33	0.022*	0.077
MMSE	−0.34	0.017*	0.077
HAMA	−0.05	0.71	0.78
HAMD	−0.08	0.59	0.78

Values are Spearman partial correlation coefficients adjusted for age. False discovery rate (FDR) correction was applied across all clinical variables. PD, Parkinson’s disease; MoCA, Montreal Cognitive Assessment; MMSE, Mini-Mental State Examination; HAMA, Hamilton Anxiety Scale; HAMD, Hamilton Depression Scale.

*P < 0.05.

Age-adjusted analyses revealed nominal negative associations between plasma α-synuclein and cognitive performance measured by MoCA and MMSE (both *P* < 0.05). These associations did not remain significant after FDR correction. No significant age-adjusted associations were observed between plasma α-synuclein and disease duration, Hoehn–Yahr stage, anxiety, or depression scores (Table 3).

In PD group, plasma α-synuclein levels were negatively correlated with age, while no significant correlations were observed with sex, disease duration, UPDRS-III score, H-Y stage, MoCA score, MMSE score, HAMA score, or HAMD score (Table 4).

**TABLE 4:** Multiple linear regression of plasma α-synuclein on clinical characteristics in PD group.

Variable	Unstandardized coefficient		Standardized coefficient	*t*	*P*	Multicollinearity statistics	
	*B*	Standard Error	*Beta*			Tolerance	VIF
(Constant)	122.177	16.668		7.33	< 0.001		
Age	−0.804	0.211	−0.648	−3.81	< 0.001	0.52	1.923
Sex	2.477	2.779	0.135	0.891	0.379	0.653	1.532
Disease duration	0.016	0.029	0.075	0.558	0.58	0.835	1.198
UPDRS-III	−0.018	0.12	−0.029	−0.15	0.881	0.398	2.512
H-Y	0.569	2.193	0.045	0.26	0.797	0.509	1.965
MoCa	0.167	0.49	0.075	0.34	0.736	0.307	3.257
MMSE	−0.926	0.536	−0.349	−1.728	0.092	0.369	2.707
HAMA	0.127	0.357	0.111	0.357	0.723	0.157	6.363
HAMD	−0.32	0.434	−0.218	−0.737	0.466	0.172	5.826
F	3.163						
Adjusted *R*^2^	0.293						
D-W	2.165						

### 3.3 Bilateral QSM: associations with clinical features and α-synuclein (PD only)

**FIGURE 4:**
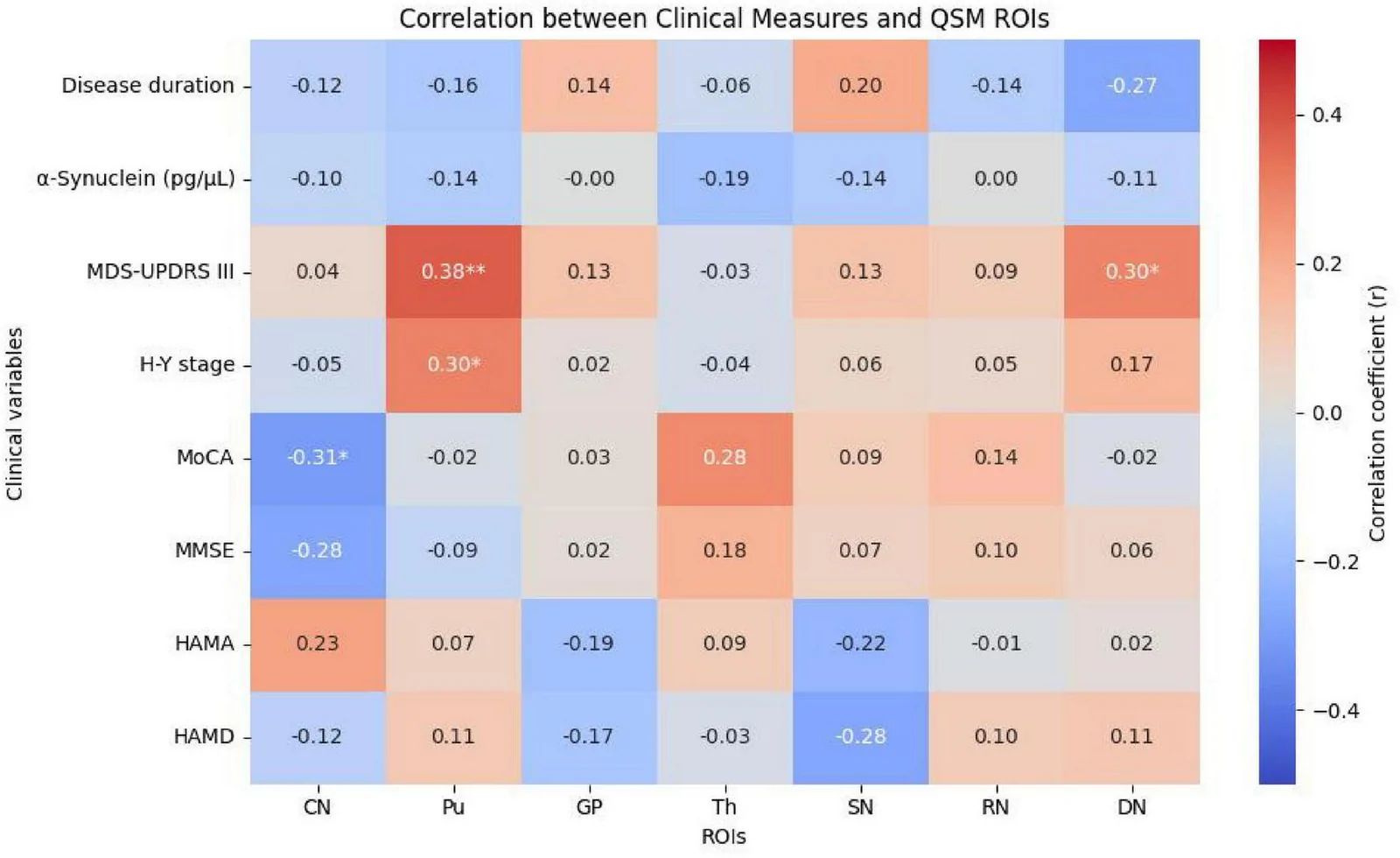
Heatmap of correlations between QSM-derived magnetic susceptibility across seven deep gray-matter ROIs and clinical measures in the PD group. Correlation coefficients (r) are displayed within each cell, with significance indicated by asterisks (*P < 0.05; **P < 0.01). Note: no associations survived FDR correction for multiple comparisons.

Raw Spearman analyses indicated nominal associations between putamen susceptibility and motor severity (UPDRS-III)(*r* = 0.381, *P* = 0.008), H-Y stage (*r* = 0.302, *P* = 0.039), between caudate nucleus susceptibility and cognitive performance (MoCA) (*r* = −0.308, *P* = 0.037), and between dentate nucleus susceptibility and motor severity (UPDRS-III) (*r* = 0.300, *P* = 0.040) (all uncorrected *P* < 0.05) (see Figure 4).

After adjustment for age, caudate nucleus susceptibility and cognitive performance (MoCA) (*r* = −0.309, *P* = 0.039), all other associations were attenuated and did not reach statistical significance. Furthermore, following FDR correction for multiple ROI comparisons, no bilateral QSM–clinical associations remained significant. Across all seven ROIs, no significant associations were observed between QSM measures and plasma α-synuclein, either before or after age adjustment and FDR correction (see Supplementary Figures 1, 2).

Taken together, although several unadjusted associations between regional susceptibility and clinical measures were observed, none remained significant after accounting for age and multiple comparisons, indicating that these findings should be interpreted cautiously.

### 3.4 Unilateral QSM in patients with lateralized symptoms

Among 25 PD patients with clearly lateralized motor symptoms (onset side matching the current more-affected side):

Contralateral Pu susceptibility positively correlated with MDS-UPDRS III (*r* = 0.510, *P* = 0.009).

**FIGURE 5:**
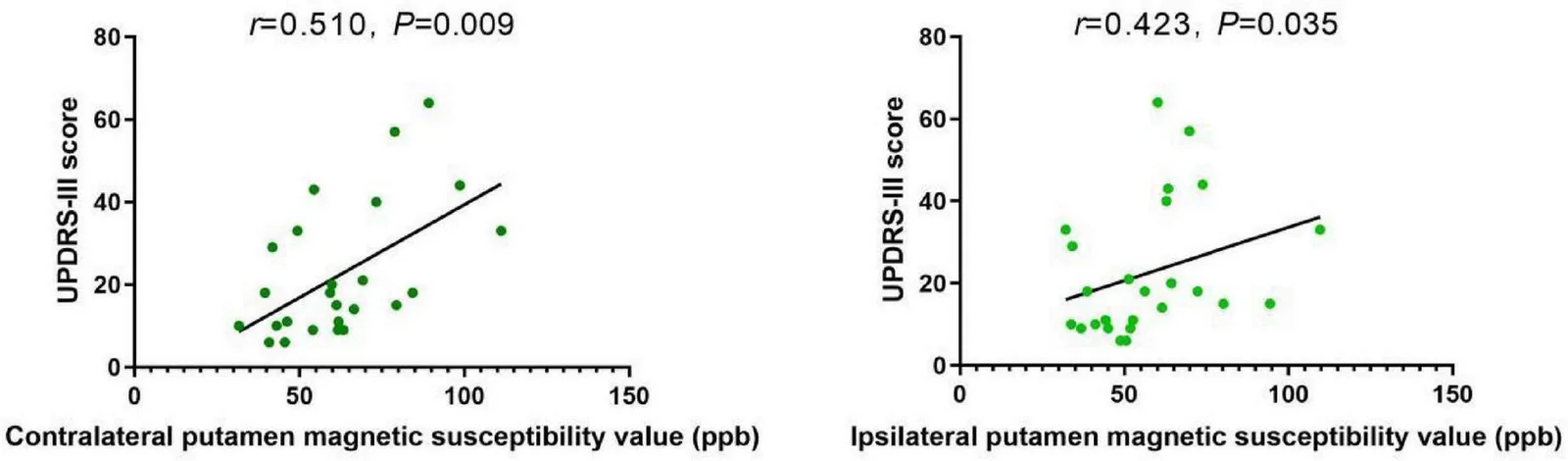
Correlation between putamen magnetic susceptibility values on the more-affected and less-affected sides and MDS-UPDRS III scores.

Ipsilateral Pu susceptibility positively correlated with MDS-UPDRS III (*r* = 0.423, *P* = 0.035) (Figure 5).

Contralateral SN susceptibility showed a negative trend with plasma α-synuclein (*r* = −0.365, *P* = 0.079), not statistically significant.

No other unilateral ROI–clinical correlations reached significance; no hemispheric differences in QSM values were detected across ROIs (*P* > 0.05). After age adjustment, only Contralateral Pu susceptibility positively correlated with MDS-UPDRS III (*r* = 0.449, *P* = 0.028),and correction for multiple comparisons, none of these unilateral associations remained statistically significant.

### 3.5 Motor subtypes: clinical comparisons

Among PD patients, 42 (80.8%) were classified as tremor-dominant (TD), 9 (17.3%) as postural instability/gait difficulty (PIGD), and 1 (1.9%) as indeterminate. Compared with the TD group, the PIGD group exhibited higher Hoehn–Yahr stage and higher anxiety and depression scores (all *P* < 0.05). There were no significant differences in age, sex, disease duration, motor severity, cognitive scores, plasma α-synuclein levels, or bilateral QSM measures between subtypes (Table 5).

**TABLE 5:** Comparison of clinical characteristics among Parkinson’s disease patients with different motor subtypes.

Variable	TD group (*n* = 42)	PIGD group (*n* = 9)	*Z/χ* ^2^	*P*
Age (years)	64.50 (62.00, 69.25)	71.00 (65.00, 73.00)	−1.934^c^	0.053
Gender/n (%)				
Male	15 (35.71)	3 (33.33)	0.000^d^	1.000
Female	27 (64.29)	6 (66.67)		
Disease duration (months)	30 (12, 60)	24 (9, 66)	−0.326^c^	0.745
MDS-UPDRS III	16.00 (9.00, 24.00)	21.00 (10.50, 43.00)	−1.226^c^	0.220
H–Y stage	2.00 (2.00, 2.00)	2.50 (1.75, 4.00)	−2.403^c^	0.016*
MoCA	20.00 (16.00, 22.00)	19.00 (12.00, 21.50)	−1.035^c^	0.301
MMSE	23.00 (21.00, 24.00)	22.00 (18.50, 24.00)	−0.959^c^	0.337
HAMA	7.00 (3.00, 12.00)	15.00 (11.50, 24.00)	−2.524^c^	0.012*
HAMD	2.00 (1.00, 7.75)	10.00 (6.00, 13.50)	−2.845^c^	0.004**

^c^ Wilcoxon rank-sum test;

^d^ Chi-square test;

*P < 0.05;

**P < 0.01.

### 3.6 Subtype-specific QSM and plasma α-synuclein

No differences were observed between TD and PIGD in plasma α-synuclein or bilateral mean QSM across ROIs (*P* > 0.05; Table 6).

**TABLE 6:** Comparison of bilateral mean QSM values of different brain nuclei and plasma α-synuclein levels among PD patients with different motor subtypes.

Variable	TD group (*n* = 42)	PIGD group (*n* = 9)	*t/Z*	*P*
α-Synuclein concentration (pg/μL)	54.92 (48.85, 59.52)	46.73 (44.65, 61.67)	−1.061^f^	0.289
CN (ppb)	42.80 (38.75, 47.78)	40.70 (35.70, 47.30)	−0.662^f^	0.508
Pu (ppb)	55.15 (45.75, 63.88)	52.20 (40.00, 75.05)	−0.054^f^	0.957
GP (ppb)	101.28 ± 26.09	88.71 ± 32.71	1.238^e^	0.222
Th (ppb)	7.90 (4.00, 10.23)	7.10 (5.93, 9.25)	−0.203^f^	0.839
SN (ppb)	116.18 ± 23.20	103.88 ± 18.72	1.477^e^	0.147
RN (ppb)	109.73 ± 18.64	98.11 ± 23.76	1.596^e^	0.118
DN (ppb)	86.31 ± 20.77	78.36 ± 23.74	1.006^e^	0.320

^e^ *t*-test;

^f^ Wilcoxon rank-sum test.

#### 3.6.1 Within-group correlations

TD group: RN with HAMD (*r* = 0.339, *P* = 0.043); Pu with MDS-UPDRS III (*r* = 0.329, *P* = 0.047); DN with MDS-UPDRS III (*r* = 0.443, *P* = 0.006) and with H–Y (*r* = 0.342, *P* = 0.038) (Supplementary Figure 3).

After age adjustment, only DN with MDS-UPDRS III (*r* = 0.388, *P* = 0.020).

PIGD group: plasma α-synuclein with MMSE (*r* = −0.698, 95% CI:−0.934 to −0.039, *P* = 0.037); SN with MDS-UPDRS III (*r* = 0.678, 95% CI: 0.001–0.929, *P* = 0.045) (Supplementary Figures 4, 5).

After age adjustment, only plasma α-synuclein with MMSE remained nominally significant (*r* = −0.715, 95% CI: −0.981 to 0.321, *P* = 0.046).

However, none of these associations remained significant after age adjustment and FDR correction. Given the very small sample size of the PIGD subgroup (*n* = 9), these findings should be interpreted with particular caution. The wide confidence intervals—particularly those crossing zero (e.g., age-adjusted plasma α-synuclein with MMSE: 95% CI −0.981 to 0.321)—reflect the inherent instability of correlation estimates in such a limited sample. Accordingly, these results are considered hypothesis-generating and warrant confirmation in larger, independent cohorts.

## 4 Discussion

In this cross-sectional study integrating peripheral and neuroimaging biomarkers, we examined how plasma α-synuclein and QSM-derived magnetic susceptibility relate to clinical heterogeneity in Parkinson’s disease. Consistent with prior meta-analyses, our data confirmed that PD patients exhibit markedly higher plasma α-synuclein concentrations than age-matched controls (Shu et al., 2024; Zubelzu et al., 2022; Duran et al., 2010; Lin et al., 2017; Lin et al., 2020; Lee et al., 2006; Youssef et al., 2021; Wang et al., 2019). This elevation supports the notion that peripheral α-synuclein reflects neuronal degeneration and abnormal protein trafficking from the central nervous system (CNS) into the circulation (Miller et al., 2004; Tokuda et al., 2006; Li et al., 2007; Lee et al., 2005; Shi et al., 2010). Similar to Lin et al. (2017) 2020, who applied SQUID-IMR technology and demonstrated diagnostic differentiation between PD and controls, we observed a high diagnostic accuracy (AUC = 0.976) using ELISA-based quantification. Interestingly, despite this strong discriminatory power, α-synuclein correlated only weakly and inversely with MDS-UPDRS-III scores. This inverse pattern, also noted by Malec-Litwinowicz et al. (2015), may indicate that as PD advances, α-synuclein becomes increasingly sequestered into insoluble aggregates (Lewy bodies), reducing its detectable soluble fraction in plasma. It is important to note that, because QSM data were unavailable for healthy controls, our analyses of magnetic susceptibility were confined to within-PD correlations. Therefore, these findings should be interpreted as exploratory regarding brain iron distribution in PD rather than as evidence of absolute iron elevation.

Differences among studies regarding plasma α-synuclein likely arise from heterogeneous sample preparation, antibody specificity, and biological variability linked to inclusion body dynamics (Kopito, 2000; McNaught et al., 2002; Ardley et al., 2003; Taylor et al., 2003; Parnetti et al., 2016). The formation of intracellular aggregates may initially serve a protective sequestration role, isolating misfolded α-synuclein from cytoplasmic targets before clearance via autophagic pathways (Gao et al., 2015; Lleó et al., 2015; Kang et al., 2013; Compta et al., 2015). However, progressive proteostasis failure can drive toxic accumulation, neuronal loss, and compensatory extracellular release (Bates and Zheng, 2014; Shi et al., 2014; Korff et al., 2013; Sui et al., 2014; Chen et al., 2020). These molecular dynamics may converge with iron-mediated oxidative stress to exacerbate neuronal injury. Moreover, α-synuclein can bidirectionally cross the blood–brain barrier (BBB), implying that peripheral elevations might mirror central overproduction or leakage secondary to neuroinflammation and BBB disruption (Chan et al., 2016; Zuo et al., 2017a; Zhu et al., 2025; Ding et al., 2017). This mechanistic interface aligns with our finding that plasma α-synuclein did not linearly reflect QSM-derived brain iron levels, suggesting distinct yet interactive systemic and neural compartments in PD pathology.

We found that plasma α-synuclein was inversely correlated with global motor severity and, within the PIGD subgroup, negatively correlated with MMSE scores, consistent with the cognitive vulnerability previously reported for this phenotype (Zuo et al., 2017a,b,c; Zhu et al., 2025; Ding et al., 2017). Notably, this inverse pattern between plasma α-synuclein and motor severity has also been observed in other cohorts. Malec-Litwinowicz et al. (2015) reported that plasma α-synuclein levels correlated inversely with motor symptom severity, consistent with our findings. Similarly, Ding et al. (2017) investigated 84 Parkinson’s disease patients and found a significant negative correlation between plasma α-synuclein and Tinetti gait score severity (*r* = −0.355, *P* < 0.001), which remained significant after multivariate adjustment (*P* = 0.002). However, the literature is not entirely consistent: Wang et al. (2019) reported a positive correlation between plasma α-synuclein and UPDRS motor scores, while Lee et al. (2006) and Zhao et al. (2022) found no significant association between plasma α-synuclein and motor severity. These discrepancies likely reflect differences in assay methods, sample processing, disease stage, and subtype composition across studies. Prior studies using ultra-sensitive immunomagnetic reduction assays have shown that elevated plasma α-synuclein associates with reduced MMSE and altered Aβ-42/Aβ-40 ratios, implicating shared amyloid and synuclein mechanisms in PD-related cognitive decline (Lin et al., 2017; Chen et al., 2020; Chan et al., 2016). Nevertheless, the observation of an inverse pattern in multiple independent cohorts—including the present study—supports the hypothesis that peripheral α-synuclein levels may decline as the disease progresses, possibly due to increased sequestration into insoluble aggregates at later stages. The attenuation of this correlation after age adjustment further suggests that age may act as a significant confounder in this relationship. Conversely, the lack of correlation with H–Y stage or disease duration suggests that peripheral α-synuclein elevation may peak during early or compensatory phases, declining as neuronal loss and inclusion formation dominate later stages (Duran et al., 2010; Lin et al., 2020). Thus, α-synuclein may serve as an early biomarker rather than a linear disease-severity index.

The AUC of 0.976 with 100% sensitivity for plasma total α-synuclein in distinguishing PD from controls exceeds the pooled estimates from recent meta-analyses (Zubelzu et al., 2022; Shu et al., 2024). This discrepancy warrants careful consideration. On the one hand, the kit we used is a clinically registered product (NMPA-approved), which provides a certain degree of manufacturing standardization and quality control compared with some research-grade kits that may vary substantially across batches. On the other hand, it is important to acknowledge several factors that may limit direct comparisons with prior studies. First, the raw readout of this ELISA kit is optical density (OD). We converted OD values to relative concentrations (pg/μL) via a laboratory-established standard curve for correlational analyses; accordingly, these values should be interpreted as relative estimates rather than absolute quantitative measures. Second, the kit’s analytical sensitivity (limit of detection) is not specified by the manufacturer in concentration units, which limits our ability to benchmark its analytical performance against other assays. Third, our study used plasma, whereas many previous studies have used serum, and the choice of blood matrix is known to influence α-synuclein measurements. Fourth, differences in cohort characteristics—including disease stage, sample size, and population genetic background—between our study and previous meta-analyses may also contribute to the observed variation in diagnostic performance. Therefore, while our findings support the potential utility of plasma α-synuclein as a PD biomarker, the specific cut-off value (41.00 pg/μL) and the high performance metrics require external validation in independent, multicenter cohorts using standardized assays before they can be generalized to broader clinical settings.

Previous quantitative susceptibility mapping studies have consistently reported increased iron deposition in deep gray-matter nuclei in Parkinson’s disease relative to healthy controls, including the putamen, globus pallidus, substantia nigra, red nucleus, and dentate nucleus. In the present study, QSM analyses were restricted to the PD cohort because susceptibility data were not available for controls; therefore, no direct between-group comparisons of regional susceptibility were performed (Wang et al., 2016; Sjöström et al., 2017; Thomas et al., 2024; Guan et al., 2017; Zhang et al., 2019; Yao et al., 2017; Chen et al., 2023; An et al., 2018). We observed that putaminal and dentate iron burden correlated positively with motor impairment (MDS-UPDRS-III) and H–Y stage, while caudate susceptibility was inversely related to cognitive scores (MoCA). These findings reinforce reports that dopaminergic depletion in the dorsal striatum parallels motor disability (Kordower et al., 2013; Wang et al., 2016; Thomas et al., 2024) and that caudate iron accumulation contributes to executive and memory decline (Mendez et al., 1989; Apostolova et al., 2010; Uchida et al., 2019). In tremor-dominant PD, the association between red-nucleus susceptibility and HAMD scores suggests that early iron accumulation in brainstem nuclei may modulate both tremor and affective symptoms (Zhang et al., 2019; Yao et al., 2017; Chen et al., 2023). Likewise, the positive correlation between substantia nigra iron and MDS-UPDRS-III in PIGD aligns with prior evidence linking nigral QSM metrics to advanced disease stages (Thomas et al., 2024; Guan et al., 2017).

However, after applying rigorous age adjustment and FDR correction for multiple comparisons across seven ROIs and eight clinical variables (section 3.4), Although several nominal associations between regional susceptibility and clinical measures were observed, none survived age adjustment and FDR correction. These findings highlight the importance of rigorous statistical control in multi-ROI neuroimaging analyses and suggest that previously reported associations may be sensitive to confounding by age and multiple testing.

Taken together, our findings suggest that while QSM provides a sensitive *in vivo* measure of regional magnetic susceptibility, its cross-sectional associations with clinical severity are modest and highly sensitive to confounding by age and multiple testing. After rigorous statistical correction, no QSM–clinical associations remained significant, underscoring the need for cautious interpretation of unadjusted findings and highlighting the importance of longitudinal designs to clarify the temporal relevance of iron accumulation in Parkinson’s disease.

Several limitations should be noted. First and foremost, QSM data were not acquired in healthy controls, which precludes direct comparisons of absolute magnetic susceptibility between PD patients and normal aging populations. Consequently, our QSM findings are strictly interpreted as exploratory within-cohort correlational analyses rather than as definitive evidence of disease-related iron elevation. Second, the sample size—particularly in the PIGD subgroup (*n* = 9)—was modest, which substantially reduces statistical power and increases the vulnerability of correlation estimates to individual data points. This is reflected in the wide confidence intervals observed for PIGD-specific correlations, some of which crossed zero despite nominal statistical significance. Therefore, these findings should be treated as hypothesis-generating rather than confirmatory. Third, the inverse correlation between plasma α-synuclein and motor severity observed in unadjusted analyses was attenuated after adjustment for age, underscoring the importance of considering age as a potential confounder in biomarker–clinical correlation studies. Fourth, the cross-sectional design precludes inferences about longitudinal trajectories of α-synuclein or iron accumulation. Fifth, we measured total plasma α-synuclein only; more specific proteoforms such as oligomeric or phosphorylated α-synuclein may show different clinical and imaging relationships. Sixth, QSM values were derived from manually drawn ROIs, which introduces some subjectivity; we attempted to minimize this by using multiple raters, multiple slices, and bilateral averaging, but automated segmentation could improve reproducibility in future studies. Seventh, we did not formally test combined diagnostic models of α-synuclein and QSM metrics; therefore, conclusions about multimodal diagnostic utility remain preliminary. Additionally, residual platelets in plasma may affect the measured α-synuclein levels. Future studies using platelet-poor plasma would help isolate the neuron-derived α-synuclein component and further clarify the specificity of the peripheral signal. Future longitudinal, multicenter studies integrating plasma, cerebrospinal, and QSM biomarkers—with automated segmentation and proteoform-specific assays—will be essential to establish reliable composite indicators for early PD detection and monitoring (Fu et al., 2021; Lancione et al., 2022; Thomas et al., 2024).

## 5 Conclusion

This study demonstrates that plasma α-synuclein levels are markedly elevated in Parkinson’s disease and exhibit strong diagnostic performance, supporting their role as a peripheral biomarker of disease presence. Quantitative susceptibility mapping revealed region-specific patterns of magnetic susceptibility within the Parkinson’s disease cohort; however, after adjustment for age and correction for multiple comparisons, no robust associations between regional susceptibility and clinical measures were identified.

Importantly, plasma α-synuclein levels and QSM-derived susceptibility measures did not show a direct association, suggesting that peripheral proteostatic dysregulation and central iron accumulation may reflect partially independent pathological processes. While these biomarkers provide complementary biological information, their combined diagnostic or prognostic utility requires confirmation in larger, longitudinal studies incorporating automated segmentation and proteoform-specific assays.

## Data Availability

The original contributions presented in the study are included in the article/Supplementary material, further inquiries can be directed to the corresponding author.
