# Supplementary material for: Plasma α-synuclein and QSM-derived susceptibility in Parkinson’s disease: associations with clinical features
Source: Front Aging Neurosci. 2026 Aug 14;18:1740309. doi: 10.3389/fnagi.2026.1740309 (PMC13521921; doi:10.3389/fnagi.2026.1740309)
Supplement: Supplementary file 1 [file Data_Sheet_1.docx]

Supplemanatry materials

Supp Fig. 1 Heatmap showing age-adjusted Spearman correlation coefficients between bilateral mean quantitative susceptibility mapping (QSM) values across seven regions of interest (ROIs) and clinical characteristics in PD patients. Warmer colors indicate positive correlations, and cooler colors indicate negative correlations. No associations survived false discovery rate correction for multiple comparisons.

|  |  |
| --- | --- |
|  |  |

Supp Fig. 2 Correlation Between Bilateral Mean QSM Values of Different Brain Nuclei and Clinical Characteristics in the PD Group.

### Supp Fig. 3 Correlation Between Mean Magnetic Susceptibility Value of the Red Nucleus and HAMD Score in the TD Group

### Supp Fig. 4 Correlation Between Plasma α-Synuclein Concentration and MMSE Score in the PIGD Group

### Supp Fig. 5 Correlation Between Mean Magnetic Susceptibility Value of the Substantia Nigra and UPDRS-III Score in the PIGD Group.
